# The Risk of Venous Thromboembolism in Patients with Gallstones

**DOI:** 10.3390/ijerph17082930

**Published:** 2020-04-23

**Authors:** Chien-Hua Chen, Cheng-Li Lin, Chia-Hung Kao

**Affiliations:** 1 Digestive Disease Center, Changbing Show-Chwan Memorial Hospital, Lukang Township, Changhua County 505, Taiwan; showchench@yahoo.com.tw; 2 Department of Food Science and Technology, Hungkuang University, Taichung 433, Taiwan; 3 Chung Chou University of Science and Technology, Yuanlin Township, Changhua County 510, Taiwan; 4 Management Office for Health Data, China Medical University Hospital, Taichung 404, Taiwan; orangechengli@gmail.com; 5 College of Medicine, China Medical University, Taichung 404, Taiwan; 6 Graduate Institute of Clinical Medical Science, School of Medicine, College of Medicine, China Medical University, Taichung 404, Taiwan; 7 Department of Nuclear Medicine and PET Center, China Medical University Hospital, Taichung 404, Taiwan; 8 Center of Augmented Intelligence in Healthcare, China Medical University Hospital, Taichung 404, Taiwan; 9 Department of Bioinformatics and Medical Engineering, Asia University, Taichung 413, Taiwan

**Keywords:** venous thromboembolism, deep vein thrombosis, pulmonary embolism, cholecystectomy

## Abstract

The objective of this study is to assess the relationship between gallstones and venous thromboembolism (VTE), including deep vein thrombosis (DVT) and pulmonary embolism (PE), and the risk of VTE after cholecystectomy for gallstones. This nationwide population-based cohort study retrieved the hospitalization database from the Longitudinal Health Insurance Research Database (LHID2000), a database belonging to the National Health Insurance (NHI) program of Taiwan. A total of 345,793 patients aged ≥ 18 years with gallstones diagnosed between 2000 and 2010 were identified as the study cohort. The beneficiaries without gallstones were randomly selected as the control cohort by propensity score matching with the study cohort at a 1:1 ratio based on age, sex, urbanization, occupation, comorbidities, and year of the index date. We compared the risk of VTE between both cohorts and measured the risk differences of VTE between the gallstones patients with (*n* = 194,187) and without cholecystectomy (*n* = 151,606). Each patient was examined from the index date until the occurrence of DVT or PE, death or withdrawal from the NHI program, or the end of 2011. The incidence rate of DVT was 7.94/10,000 person-years for the non-gallstones cohort and 9.64/10,000 person-years for the gallstones cohort (hazard ratio (HR) = 1.35, 95% confidence interval (CI) = 1.25–1.47), respectively (*p* < 0.001). The incidence rate of PE was 3.92/10,000 person-years for the non-gallstones cohort and 4.65/10,000 person-years for the gallstones cohort (HR = 1.35, 95% CI = 1.20–1.53), respectively (*p* < 0.001). The cumulative incidence of DVT (6.54/10,000 person-years vs 14.6/10,000 person-years, adjusted hazard ratio (aHR) = 0.60, 95% CI = 0.54–0.67) and PE (3.29/10,000 person-years vs 6.84/10,000 person-years, aHR = 0.67, 95% CI = 0.58–0.77) for gallstones patients was lower in the cholecystectomy cohort than that in the non-cholecystectomy cohort after adjustment for age, sex, urbanization level, occupation, frequency of medical visits, history of pregnancy, and comorbidities (log-rank test, *p* < 0.001). Our findings indicate that the risk of DVT or PE in patients with gallstones was greater than those without gallstones. However, the risk of DVT and PE in the patients with gallstones would decrease after cholecystectomy. This area of research needs more studies to ascertain the pathogenesis for the contribution of gallstones to the development of VTE and the protective mechanisms of cholecystectomy against the development of VTE.

## 1. Introduction 

Venous thromboembolism (VTE) mainly consists of deep vein thrombosis (DVT) and pulmonary embolism (PE). DVT can predispose to the development of PE and the leg is the most common location of DVT, although the arm, splanchnic vein, and the cerebral vein can also be involved. Next to acute myocardial infarction and stroke, VTE ranks as the third most common vascular disease globally [1]. The incidence of VTE varies with ethnicity with a greatest annual incidence of 71–117/100,000 persons in Caucasian populations; nevertheless, the incidence of VTE is lower in Asian populations and the reported incidence of Taiwan is 15.9/100,000 person-years [2,3,4,5]. VTE leads to a substantial socioeconomic disability and casts a heavy burden on medical expenditures due to long-term post-thrombotic syndrome [6,7]. However, the provoking factors cannot be identified in approximately 1/3–1/2 of VTE episodes, and therefore it is important to identify specific risk factors for the prevention of VTE [8]. 

Gallstone disease is the leading cause for hospitalization in the gastrointestinal department, and the reported prevalence is 10%–15% in the United States and 5% in Taiwan [9,10,11,12]. The reported annual incidence of gallstones complications is approximately 1%–2%, but the number of cholecystectomies has been discrepantly outnumbered after the introduction of laparoscopic cholecystectomy due to its relatively less invasiveness, lower surgical risk, and better patients’ acceptance [13]. Oxidative stress, which has been proposed a possible causative factor of VTE, has been shown to be closely associated with the number of stones and cholecystitis in gallstones disease [14,15]. Furthermore, VTE is reportedly related to atherosclerosis in a case–control study and the possible explanations may be shared risk factors or common pathogenesis due to oxidative stress [16,17]. Gallstone disease has been reportedly associated with the development of cardiovascular diseases (CVD), a thromboembolic arterial disease; however, the association between gallstones and VTE, a thromboembolic venous disease, has never been mentioned in the literature [18].

In this study, we hypothesize that gallstone disease is associated with an increased risk of VTE and the risk may decrease if gallstone disease per se is a risk factor of VTE, rather than an epiphenomenon. We conduct a nationwide population-based cohort study by analyzing data from the Longitudinal Health Insurance Research Database (LHID2000) of the Taiwan National Health Insurance Research Database (NHIRD) to determine the association between gallstones and the subsequent development of VTE, including DVT and PE. Furthermore, we also assess the effect of cholecystectomy on the development of VTE in patients with gallstones.

## 2. Methods

### 2.1. Data Source

The LHID2000 of the NHIRD comprised the database for inpatient claims in the National Health Insurance (NHI) program of Taiwan and we used this database to conduct this retrospective nationwide cohort study [19]. The mandatory NHI program of Taiwan has been launched since 1 March 1995 and this unique health care system, a government-owned single payer system, has covered approximately 99.2% of the 23.74 million residents of Taiwan [20,21]. All the citizens could fairly access the healthcare system without discrimination. The finance of NHI was mainly supported by copayment in each medical consultation, the premiums levied according to payroll tax, and the government funding. To mitigate and contain the increasing medical expenditures, the NHI program has changed the payment system from fee-for-service to a prospective global budget based on the expected annual gross domestic product since 2002. For medical reimbursement, the government quarterly audited all insurance claims according to the standard diagnostic criteria. In this study, we defined the claim codes based on the 2001 International Classification of Diseases, Ninth revision, Clinical Modification (ICD-9-CM). 

### 2.2. Ethics Statement

The database of NHIRD was administered by the National Health Research Institute (NHRI) of Taiwan, a non-profit organization for medical research, and the personal information has been encrypted to protect privacy and to provide the researchers with the relevant claims information after a formal application to NHRI without the requirement of patient consent. The Institutional Review Board (IRB) of China Medical University (CMUH104-REC2-115-CR4) has approved this study.

### 2.3. Sampled Patients

This study identified patients aged ≥ 18 years with newly diagnosed gallstones (ICD-9-CM 574) between 1 January 2000, and 31 December 2010. The control cohort randomly selected patients without a history of gallstones or cholecystectomy by 1:1 propensity score matching with the case cohort based on age group (every 5-year span), sex, occupation category, urbanization level, history of pregnancy, frequency of medical visits, comorbidities of atrial fibrillation, hypertension, diabetes, cerebrovascular accident (CVA), heart failure, lower leg fracture or surgery, all cancers, obesity, alcohol-related illness and chronic obstructive pulmonary disease (COPD), and the index date for the diagnosis of gallstones. The reference index date for the control patients was randomly appointed based on the month and day with the same index year of the matched patients. Moreover, the patients with gallstones were divided into two groups according to their history of cholecystectomy or not. Patients with a history of DVT (ICD-9-CM 453.8) or PE (ICD-9-CM 415.1) and those with incomplete information on age or sex at the baseline were excluded from the cohorts. Each patient was examined from the index date until the occurrence of DVT or PE, death or withdrawal from the NHI program due to emigration out of Taiwan, or the end of 2011. However, the diagnosis of iatrogenic PE (ICD-9-CM 415.11) was excluded from the endpoints. Both identified cause-specific and non-cause-specific deaths were included in the analysis, but the deaths with undetermined causes would be censored. The urbanization levels were classified according to the population density (people/km^2^), proportion of residents with a higher education, proportion of elderly and agricultural population, and the number of physicians per 100,000 people in each area [22]. White collar occupations consisted of indoor workers such as public servants, educators, or administrative personnel in business and industries. Blue collar occupations consisted of outdoor workers with longer hours such as fishermen, farmers, or industrial laborers. The individuals who were retired, unemployed, or working for subsidy were classified as those with other occupations.

### 2.4. Statistical Analysis

We compared the distribution of the categorical variables by the chi-squared test and the continuous variables by the Student’s *t* test, respectively. We used the Kaplan–Meier method to compare the cumulative incidence of VTE, including DVT and PE, and survival between the cohorts; nevertheless, the log-rank test was used to examine the differences. The incidence density rates of DVT and PE were measured by dividing the number of DVT and PE events by the number of person-years for each risk factor, and the results were then stratified by age, sex, occupation, urbanization, and presence or absence of a comorbidity or the history of pregnancy. With the hazard ratios (HRs) and 95% confidence intervals (CIs) estimated, a univariable Cox proportional hazard regression model was used to assess the risk of DVT and PE associated with gallstones. However, we used univariable and multivariable Cox proportional hazard regression models to assess the risk of DVT and PE associated with cholecystectomy. The adjusted hazard ratios (aHRs) and 95% confidence intervals (CIs) were estimated by using the Cox model, and were adjusted for age, sex, urbanization level, occupation, frequency of medical visits, history of pregnancy and comorbidity of hypertension, diabetes, CVA, heart failure, all cancers, atrial fibrillation, lower leg fracture or surgery, obesity, alcohol-related illness, and COPD. The E-value and CI, which would add a degree of quantification to possible confounding, were computed [23].

All the data were analyzed by utilizing SAS Version 9.4 (SAS Institute, Cary, NC, USA), and a two-tailed *p* < 0.05 was considered as statistically significant. 

## 3. Results

The study identified a gallstones cohort of 345,793 patients and a non-gallstones cohort of 345,793 patients (Table 1). The gallstones and non-gallstones cohorts were well matched for age, sex, urbanization level, occupation, frequency of medical visits, history of pregnancy, and comorbidities. The mean ages in the gallstones and non-gallstones cohorts were 60.6 ± 16.7 and 60.5 ± 16.6 years, respectively. The gallstones patients had an equivalent distribution between women (50.4%) and men (49.6%), and most patients were older than 65 years (44.3%). The top three common comorbidities in the gallstones cohort were hypertension (32.8%), diabetes (21.1%), and COPD (7.4%), in that order of frequency. Among the 345,793 gallstones patients, 194,187 had a history of cholecystectomy and 151,606 did not have a history of cholecystectomy. The non-cholecystectomy cohort had greater rates of the population aged ≥ 65 years, frequency of medical visits, and comorbidities; nevertheless, the cholecystectomy cohort had greater rates of women and history of pregnancy.

Table 2 presents the comparison of the incidence densities of VTE, including DVT and PE, between patients with and without gallstones based on the stratification of demographic characteristics and the presence or absence of a comorbidity or history of pregnancy. Compared with the non-gallstones cohort, the gallstones cohort had greater risks of VTE, including either DVT or PE. The overall incidence rate of VTE in the non-gallstones and gallstones cohorts were 11.2 and 13.6 per 10,000 person-years, respectively (*p* < 0.001). The incidence rate of DVT was 7.94 per 10,000 person-years for the non-gallstones cohort and 9.64 per 10,000 person-years for the gallstones cohort (HR (hazard ratio) = 1.35, 95% confidence interval (CI) = 1.25–1.47), respectively (*p* < 0.001). The incidence rate of PE was 3.92 per 10,000 person-years for the non-gallstones cohort and 4.65 per 10,000 person-years for the gallstones cohort (HR = 1.35, 95% CI = 1.20–1.53), respectively (*p* < 0.001). The E-value = 2.04 for the estimate, implying that unmeasured confounding was likely. The overall incidence density rates of VTE, DVT, and PE in the non-cholecystectomy group were 20.5 per 10,1000 person-years, 14.6 per 10,000 person-years, and 6.84 per 10,000 person-years, respectively. Notably, the overall incidence rates of VTE, DVT, and PE in the gallstones patients with cholecystectomy decreased to be 9.27 per 10,000 person-years, 6.54 per 10,000 person-years, and 3.29 per 10,000 person-years, respectively. The absolute risk reduction of VTE after cholecystectomy for gallstones was the experimental event rate (20.5/10,000) and the control event rate (9.27/10,000) = 11.23/10,000, and the number needed to treat (1/ARR) of cholecystectomy for gallstones to prevent one VTE per year was approximately 890 in our study.

**Figure 1:**
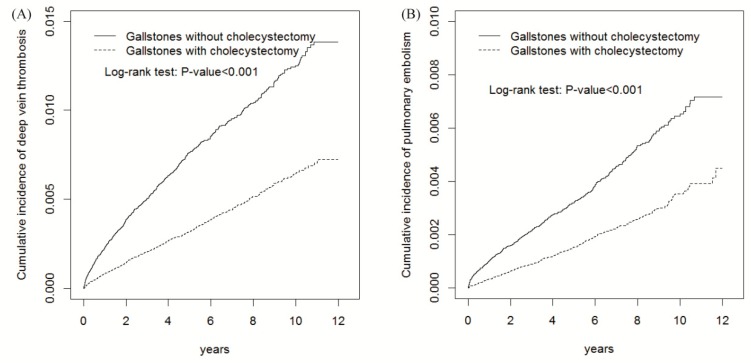
The cummulative incidence of deep vein thrombosis (DVT) and pulmonary embolism (PE) in gallstones patients with cholecystectomy and without cholecystectomy.

Figure 1 shows that the cumulative incidences of DVT and PE for gallstones were lower in the cholecystectomy cohort than those in the cholecystectomy cohort (log-rank test *p* < 0.001). The average follow-up duration for DVT was 6.20 ± 3.21 years for the non-gallstones cohort, 5.43 ± 3.27 years for the gallstones patients with cholecystectomy, and 4.30 ± 3.31 years for the gallstones patients without cholecystectomy. The average follow-up duration for PE was 6.20 ± 3.21 years for the non-gallstones cohort, 5.43 ± 3.27 years for the gallstones patients with cholecystectomy, and 4.31 ± 3.31 years for the gallstones patients without cholecystectomy. 

Table 3 presents the comparison of the incidence densities of VTE, including DVT and PE, between the gallstones patients with and without cholecystectomy based on the stratification of demographic characteristics and the presence or absence of a comorbidity or history of pregnancy. Compared with patients without cholecystectomy, those with cholecystectomy were associated with a decreased risk of VTE (aHR = 0.62, 95% CI = 0.57–0.67), including DVT (aHR = 0.60, 95% CI = 0.54–0.67) and PE (aHR = 0.67, 95% CI = 0.58–0.77), after adjustment for age, sex, urbanization level, occupation, frequency of medical visits, history of pregnancy and comorbidity of hypertension, diabetes, CVA, heart failure, all cancers, atrial fibrillation, lower leg fracture or surgery, obesity, alcohol-related illness, and COPD.

## 4. Discussion 

Cholecystectomy in clinical settings is mainly indicated for biliary complications of gallstones, such as cholecystitis, cholangitis, pancreatitis, and biliary tract cancer [13]. In this study we demonstrate that gallstones would increase the risk of VTE, including either DVT or PE, and the risk would diminish after cholecystectomy. In this way, gallstones may be beyond a biliary tract disease and be a marker of a systemic disease. Furthermore, cholecystectomy will ameliorate the risk of VTE, a potentially lethal disease. 

Our results reveal that the risk of VTE in patients with gallstones decreased after cholecystectomy after adjustment for age, sex, urbanization level, occupation, frequency of medical visits, history of pregnancy and comorbidity of hypertension, diabetes, CVA, heart failure, all cancers, atrial fibrillation, lower leg fracture or surgery, obesity, alcohol-related illness, and COPD. Moreover, cholecystectomy ameliorated the development of VTE throughout each subgroup of age, sex, urbanization level, occupation category, and the presence or absence of a comorbidity or pregnancy. In addition, the protective effect of cholecystectomy against the development of DVT and PE increased with an incremental duration of follow-up (Figure 1). These findings confirm a protective effect of cholecystectomy against the development of VTE in patients with gallstones although the mechanism and the causal relationship could not be ascertained in this observational study. 

Most clinicians recently have focused on CVD, in addition to biliary complications, attributed by gallstones [18,24,25]. However, as shown in Table 2, this study demonstrates that the risk of VTE, including either DVT or PE, in the gallstones cohort was 1.35-fold greater than that of the control cohort even though the control cohort had a longer duration of follow-up. In addition, this study finds that the risk of VTE in the gallstones patients without cholecystectomy was 1.84-fold greater, with 1.91-fold greater for DVT and 1.71-fold greater for PE, than that of the individuals without gallstones. However, as shown in Table 2 and Table 3, the risk of VTE significantly decreased to a level comparable to that of the non-gallstones cohort when the gallstones patients underwent cholecystectomy. Our findings indicate that gallstones per se may represent a risk factor of VTE, a vascular disease not limited to atherosclerosis, although we cannot exclude the possibility of gallstones as an epiphenomenon of VTE. However, cholecystectomy deserves more studies to change its clinical setting, which is mainly limited to biliary tract complications. 

More than 80% of patients with gallstones will remain asymptomatic and it is possible to misclassify these asymptomatic patients as the general population without gallstones [26]. Moreover, it will raise a concern about the surveillance bias as the patients with gallstones might access more studies to yield the diagnosis of VTE. We also compared the incidence of VTE between the clinically relevant gallstones patients with cholecystectomy and without cholecystectomy to diminish the surveillance bias and our findings consistently supported the merit of cholecystectomy against the development of VTE in patients with gallstones. Another sensitivity analysis was the validation of VTE by the assessment of not only DVT but also PE, and our findings indicated that both risks were consistently lower in the cholecystectomy patients. Notably, the case number was comparable between the gallstones with and without cholecystectomy and the explanations might be that the database of LHID2000 mainly contained the claims data for hospitalization and the thresholds for cholecystectomy declined in a laparoscopic era [13].

The exact mechanisms for the association between gallstones, cholecystectomy, and VTE cannot be determined in this study. The possible explanations for the association between gallstones and VTE may be shared risk factors between gallstones and VTE, or the common pathogenesis, oxidative stress, observed in VTE and gallstones [14,15,27]. Oxidative stress has been shown to be an important mediator for abnormal platelet aggregation and impaired endothelial vasodilatation [15,27]. Moreover, the products of the free radical reaction have a positive linear correlation with the number of stones and the severity of cholecystitis [14]. However, the possible mechanisms underlying the protective effect of cholecystectomy against the development of VTE in patients with gallstones may be the consequence of diminishing oxidative stress after cholecystectomy for gallstones or improved lifestyle modifications after cholecystectomy. Due to pneumoperitoneum and Trendelenburg positioning, it has been reported that a coagulation cascade could be activated during cholecystectomy [28]. However, it should be noted that the actual incidence of DVT was quite rare within seven days after cholecystectomy, and our end outcome of VTE was a consequence of long-time follow-ups rather than an immediate postoperative period.

There are several strengths in our study. Firstly, this is the first population-based cohort study on the association between gallstones and VTE. Furthermore, with the comparison between cholecystectomy and non-cholecystectomy cohorts to diminish the possibility of the surveillance bias, we are the first to express that the risk of VTE in gallstones will decrease after cholecystectomy. Secondly, we assess not only the risk of DVT but also that of PE to validate our endpoint of venous thromboembolism. Finally, our population-based cohort study uses an official database of the mandatory NHI Program, which covered more than 99.2% of residents in Taiwan, and provides the generalizability of the findings with a 12-year-long follow-up in Taiwan. However, it requires more international studies to ascertain whether this generalizability will vary with environment or ethnics.

There are several limitations in our study. Firstly, the major limitation of this study is the inherent unavailability of several potential confounding related to lifestyle or environment, even though we have substituted the diagnosis of obesity for body mass index, COPD for smoking, and alcohol-related illness for alcohol drinking. Secondly, the accuracy of the claim data cannot be individually reviewed. However, the Taiwanese government has audited all insurance claims according to the standard diagnostic criteria for medical reimbursement. Furthermore, the claims of common chronic diseases, medication, and medical resource utilization in NHIRD have shown a substantial concordance in the literature [29,30]. Furthermore, the claim data for cholecystectomy, DVT, and PE should be highly reliable for their definition of the diagnostic criteria. To avoid the misclassification of chronic or asymptomatic VTE into normal outcomes as much as possible, we only used ICD-9-CM 453.8 and ICD-9-CM 415.1 to enroll patients with acute DVT and PE, respectively. Thirdly, the possible skewed association between gallstones and VTE due to the surveillance bias will be minimized in assessing the association between cholecystectomy and VTE since we have compared the outcome of VTE between the clinically relevant gallstones with and without cholecystectomy. Finally, the protective mechanisms of cholecystectomy against the development of VTE cannot be ascertained in this observational study. 

In conclusion, our findings demonstrate that the risk of VTE, including either DVT or PE, is greater in the patients with gallstones and that cholecystectomy will ameliorate the risk to a degree comparable to those without gallstones. Our major limitation is the inherent inability to obtain information on several potential confounding factors in the claims database, and it needs more studies to ascertain the pathogenesis of VTE caused by gallstones, the protective mechanisms of cholecystectomy against the development of VTE, and the cost-benefit effect of prophylactic cholecystectomy against the development of VTE in gallstones patients.

### Strengths and Limitations of This Study

1. Our population-based cohort study uses an official database of National Health Insurance Program, which mandatorily covered more than 99.2% of residents in Taiwan, and provides generalized findings with a 12-year-long follow-up in Taiwan.
2. We assess not only the risk of deep vein thrombosis but also that of pulmonary embolism to validate our endpoint of venous thromboembolism.
3. The comparison between cholecystectomy and non-cholecystectomy cohorts for the development of venous thromboembolism in patients with gallstones will diminish the surveillance bias in symptomatic gallstones.
4. This study is subject to the inherent unavailability of several potential confounding factors, and the protective mechanisms of cholecystectomy against the development of venous thromboembolism cannot be ascertained in this observational study.

## 5. Conclusions 

Our findings indicate that the risk of DVT or PE in patients with gallstones was greater than those without gallstones. However, the risk of DVT and PE in the patients with gallstones would decrease after cholecystectomy. This area of research needs more studies to ascertain the pathogenesis for the contribution of gallstones to the development of VTE and the protective mechanisms of cholecystectomy against the development of VTE. 

## Figures and Tables

**Table 1:** Comparison of demographics and comorbidity between gallstones patients and controls.

Variable		Gallstones			
	Comparison (*n* = 345,793)	Total (*n* = 345,793)	Without Cholecystectomy (*n* = 151,606)	With Cholecystectomy (*n* = 194,187)	Standardized Mean Differences ^§^
	*n* (%)	*n* (%)	*n* (%)	*n* (%)	
Age, years					
≤49	97,410(28.2)	97,904(28.3)	29,558(19.5)	68,346(35.2)	0.003
50–64	95,967(27.8)	94,795(27.4)	36,390(24.0)	58,405(30.1)	0.01
≥65	152,416(44.1)	153,094(44.3)	85,658(56.5)	67,436(34.7)	0.004
Mean (SD)	60.5(16.6)	60.6(16.7)	65.3(16.2)	56.9(16.1)	0.01
Gender					
Female	173,789(50.3)	174,157(50.4)	69,306(45.7)	104,851(54.0)	0.002
Male	172,004(49.7)	171,636(49.6)	82,300(54.3)	89,336(46.0)	0.002
Urbanization level ^†^					
1 (highest)	92,837(26.9)	92,239(26.7)	35,124(23.2)	57,115(29.4)	0.004
2	102,619(29.7)	102,359(29.6)	42,707(28.2)	59,652(30.7)	0.002
3	56,317(16.3)	56,398(16.3)	24,780(16.3)	31,618(16.3)	0.001
4 (lowest)	94,020(27.2)	94,797(27.4)	48,995(32.3)	45,802(23.6)	0.01
Occupation category					
White collar	158,025(45.7)	157,439(45.5)	60,682(40.0)	96,757(49.8)	0.003
Blue collar	142,477(41.2)	142,445(41.2)	67,529(44.5)	74,916(38.6)	0.000
Others ^‡^	45,291(13.1)	45,909(13.3)	23,395(15.4)	22,514(11.6)	0.01
Frequency of medical visits/ per year Median (IQR)	12(5–39)	13(5–39)	21(8–57)	9(4–25)	0.046
Pregnancy	16,773(4.85)	17,465(5.05)	3019(1.99)	14,446(7.44)	0.01
Comorbidity					
Hypertension	114,177(33.0)	113,501(32.8)	58,732(38.7)	54,769(28.2)	0.004
Diabetes	72,235(20.9)	72,779(21.1)	39,545(26.1)	33,234(17.1)	0.004
CVA	38,184(11.0)	39,898(11.5)	25,873(17.1)	14,025(7.22)	0.02
Heart failure	16,693(4.83)	18,407(5.32)	12,462(8.22)	5945(3.06)	0.02
All cancer	21,958(6.35)	21,730(6.28)	12,132(8.00)	9598(4.94)	0.003
Atrial fibrillation	9089(2.63)	10,193(2.95)	6371(4.20)	3822(1.97)	0.02
Lower leg fracture or surgery	19,925(5.76)	21,836(6.31)	13,299(8.77)	8537(4.40)	0.02
Obesity	920(0.27)	1127(0.33)	296(0.20)	831(0.43)	0.01
Alcohol-related illness	9177(2.65)	10,557(3.05)	7506(4.95)	3051(1.57)	0.02
COPD	21,925(6.34)	25,604(7.40)	17,031(11.2)	8573(4.41)	0.04

^§^ A standardized mean difference of ≤0.10 indicates a negligible difference between the two cohorts; IQR denotes interquartile range; CVA denotes cerebral vascular disease; SD denotes standard deviations; ^†^ The urbanization level was categorized by the population density of the residential area into 4 levels, with level 1 as the most urbanized and level 4 as the least urbanized; ^‡^ Other occupations included primarily retired, unemployed, or low-income populations.

**Table 2:** Incidence and hazard ratio of venous thromboembolism (VTE), including deep vein thrombosis (DVT) and pulmonary embolism (PE), between patients with and without gallstones based on the stratification of demographic characteristics and the presence or absence of a comorbidity or history of pregnancy.

Variable	Comparison *n* = 345793		Gallstones								
			Total *n* = 345793			Without Cholecystectomy *n* = 151606			With Cholecystectomy *n* = 194187		
	Event No	Rate	Event No	Rate	HR (95% CI)	Event No	Rate	HR (95% CI)	Event No	Rate	HR (95% CI)
VTE	2403	11.2	2314	13.6	1.35(1.26, 1.45)***	1338	20.5	1.84(1.67, 2.02)***	976	9.27	0.97(0.88, 1.08)
DVT	1700	7.94	1644	9.64	1.35(1.25, 1.47)***	955	14.6	1.91(1.70, 2.14)***	689	6.54	0.94(0.84, 1.06)
Age, year											
20–49	208	2.88	211	3.75	1.44(1.12, 1.84)**	109	6.73	1.98(1.35, 2.89)***	102	2.54	1.13(0.81, 1.56)
50–64	432	6.79	383	7.57	1.27(1.06, 1.51)**	208	11.6	2.14(1.63, 2.81)***	175	5.35	0.86(0.68, 1.09)
65+	1060	13.5	1050	16.5	1.40(1.25, 1.57)***	638	20.5	1.83(1.57, 2.13)***	412	12.7	0.99(0.83, 1.17)
Gender											
Women	917	8.27	888	9.90	1.32(1.17, 1.49)***	494	16.0	1.76(1.48, 2.08)***	394	6.71	0.99(0.84, 1.17)
Men	783	7.58	756	9.35	1.35(1.19, 1.54)***	461	13.5	1.96(1.64, 2.34)***	295	6.33	0.88(0.73, 1.06)
Urbanization level^†^											
1 (highest)	416	7.06	419	8.97	1.32(1.10, 1.59)**	217	14.1	1.87(1.42, 2.48)***	202	6.45	0.99(0.77, 1.27)
2	504	7.82	479	9.36	1.38(1.16, 1.63)***	284	15.2	2.17(1.69, 2.77)***	195	6.00	0.84(0.66, 1.08)
3	295	8.47	273	9.84	1.23(0.98, 1.54)	166	15.6	1.75(1.27, 2.41)***	107	6.25	0.87(0.63, 1.19)
4 (lowest)	485	8.65	473	10.5	1.36(1.15, 1.61)***	288	14.0	1.80(1.43, 2.26)***	185	7.58	0.96(0.74, 1.23)
Occupation category											
White collar	655	6.44	638	7.96	1.39(1.20, 1.60)***	405	14.0	2.25(1.83, 2.77)***	259	4.88	0.86(0.70, 1.05)
Blue collar	775	8.96	737	10.6	1.26(1.10, 1.44)***	171	18.5	1.59(1.32, 1.91)***	332	8.20	1.00(0.83, 1.20)
Others ^‡^	270	10.4	269	12.8	1.53(1.18, 1.97)**	259	4.88	2.69(1.84, 3.93)***	98	8.33	0.86(0.60, 1.23)
Comorbidity ^§^ or history of pregnancy											
None	548	4.75	453	5.22	1.21(1.03, 1.42)*	227	8.32	1.89(1.48, 2.42)***	226	3.80	0.86(0.69, 1.06)
With any one	1152	11.7	1191	14.2	1.43(1.29, 1.58)***	728	19.2	1.78(1.55, 2.05)***	463	10.1	1.07(0.92, 1.26)
PE	841	3.92	794	4.65	1.35(1.20, 1.53)***	447	6.84	1.71(1.45, 2.03)***	347	3.29	1.05(0.88, 1.24)
Age, year											
20–49	67	0.93	96	1.70	2.12(1.40, 3.21)***	59	3.64	2.77(1.59, 4.81)***	37	0.92	1.49(0.81, 2.73)
50–64	187	2.94	170	3.36	1.29(0.98, 1.69)	80	4.47	1.61(1.06, 2.42)*	90	2.75	1.09(0.77, 1.56)
65+	587	7.47	528	8.27	1.32(1.12, 1.56)**	308	9.85	1.58(1.26, 1.98)***	220	6.75	1.06(0.83,1.36)
Gender											
Women	461	4.15	420	4.68	1.33(1.12, 1.59)**	221	7.12	1.53(1.19, 1.96)***	199	3.38	1.16(0.91, 1.49)
Men	380	3.67	374	4.62	1.40(1.15,1.69)***	226	6.58	1.99(1.53, 2.59)***	148	3.17	0.92(0.69, 1.22)
Urbanization level ^†^											
1 (highest)	200	3.39	188	4.02	1.26(0.94, 1.71)	93	6.02	1.37(0.88, 2.11)	95	3.03	1.18(0.78, 1.78)
2	239	3.71	195	3.81	1.26(0.97, 1.64)	103	5.51	1.62(1.11, 2.36)*	92	2.83	0.99(0.68, 1.43)
3	130	3.73	120	4.32	1.45(1.02, 2.07)*	63	5.91	1.73(1.05, 2.85)*	57	3.33	1.21(0.73, 1.99)
4 (lowest)	272	4.85	291	6.47	1.67(1.32, 2.11)***	188	9.14	2.53(1.82, 3.51)***	103	4.22	1.03(0.73, 1.45)
Occupation category											
White collar	312	3.07	296	3.69	1.38(1.11, 1.71)**	165	6.08	1.66(1.23, 2.25)***	131	2.47	1.12(0.82, 1.53)
Blue collar	412	4.76	395	5.69	1.46(1.21, 1.76)***	219	7.57	1.74(1.34, 2.25)***	176	4.34	1.20(0.91, 1.57)
Others ^‡^	117	4.48	103	4.89	1.40(0.92, 2.13)	63	6.78	2.40(1.28, 4.48)**	40	3.40	0.80(0.44, 1.47)
Comorbidity ^§^ or history of pregnancy											
None	259	2.24	231	2.66	1.47(1.16, 1.87)**	112	4.10	1.81(1.28, 2.58)***	119	2.00	1.21(0.87, 1.69)
With any one	582	5.88	563	6.71	1.34(1.15, 1.57)***	335	8.81	1.67(1.36, 2.06)***	228	4.97	1.02(0.81, 1.29)

Rate, per 10,000 person-years; HR: relative hazard ratio; ^†^ The urbanization level was categorized by the population density of the residential area into 4 levels, with level 1 as the most urbanized and level 4 as the least urbanized; ^‡^ Other occupations included primarily retired, unemployed, or low-income populations; ^§^ Individuals with any comorbidity of hypertension, diabetes, CVA, heart failure, all cancers, atrial fibrillation, lower leg fracture or surgery, obesity, alcohol-related illness, and COPD were classified into the comorbidity group; *p < 0.05, **p < 0.01, ***p < 0.001.

**Table 3:** Incidence and hazard ratio of VTE, including DVT and PE, between patients with and without gallstones based on the stratification of demographic characteristics and the presence or absence of a comorbidity or history of pregnancy.

Variable	Without cholecystectomy *n* = 151606	With cholecystectomy *n* = 194187
	aHR (95% CI)	aHR (95% CI)
VTE	1.00	0.62(0.57, 0.67)***
DVT	1.00	0.60(0.54, 0.67)***
Age, year		
20–49	1.00	0.56(0.42, 0.75)***
50–64	1.00	0.53(0.43, 0.65)***
65+	1.00	0.64(0.56, 0.72)***
Gender		
Women	1.00	0.63(0.55, 0.72)***
Men	1.00	0.57(0.49, 0.67)***
Urbanization level ^†^		
1 (highest)	1.00	0.68(0.56, 0.83)***
2	1.00	0.53(0.44, 0.64)***
3	1.00	0.51(0.40, 0.66)***
4 (lowest)	1.00	0.68(0.56, 0.82)***
Occupation category		
White collar	1.00	0.50(0.42, 0.59)***
Blue collar	1.00	0.74(0.63, 0.86)***
Others ^‡^	1.00	0.56(0.43, 0.72)***
Comorbidity ^§^ or history of pregnancy		
None	1.00	0.58(0.48, 0.70)***
With any one	1.00	0.60(0.53, 0.67)***
PE	1.00	0.67(0.58, 0.77)***
Age, year		
20–49	1.00	0.32(0.21, 0.50)***
50–64	1.00	0.71(0.52, 0.97)*
65+	1.00	0.74(0.62, 0.88)***
Gender		
Women	1.00	0.75(0.61, 091)**
Men	1.00	0.62(0.50, 0.76)***
Urbanization level ^†^		
1 (highest)	1.00	0.74(0.55, 1.00)*
2	1.00	0.69(0.51, 0.93)*
3	1.00	0.78(0.53, 1.13)
4 (lowest)	1.00	0.59(0.46, 0.75)***
Occupation category		
White collar	1.00	0.60(0.47, 0.76)***
Blue collar	1.00	0.76(0.62, 0.93)**
Others^‡^	1.00	0.62(0.41, 0.94)*
Comorbidity ^§^ or history of pregnancy		
None	1.00	0.60(0.46, 0.79)***
With any one	1.00	0.69(0.58, 0.82)***

Rate, per 1000 person-years; aHR: adjusted hazard ratio; multivariable analysis including age, sex, urbanization level, occupation, frequency of medical visits, history of pregnancy and comorbidity of hypertension, diabetes, CVA, heart failure, all cancers, atrial fibrillation, lower leg fracture or surgery, obesity, alcohol-related illness, and COPD; ^†^ The urbanization level was categorized by the population density of the residential area into 4 levels, with level 1 as the most urbanized and level 4 as the least urbanized. ^‡^ Other occupations included primarily retired, unemployed, or low-income populations. ^§^ Individuals with any comorbidity of hypertension, diabetes, CVA, heart failure, all cancers, atrial fibrillation, lower leg fracture or surgery, obesity, alcohol-related illness, and COPD were classified into the comorbidity group; *p < 0.05, **p < 0.01, ***p < 0.001.

## Abbreviations

VTE: venous thromboembolism; DVT: deep vein thrombosis; PE: pulmonary embolism; CVD: cardiovascular diseases; LHID2000: Longitudinal Health Insurance Research Database 2000; NHI: National Health Insurance; aHR: adjusted hazard ratio; CI: confidence interval; NHIRD, National Health Insurance Research Database; ICD-9-CM: International Classification of Diseases, Ninth Edition, Clinical Modification; NHRI: National Health Research Institute; IRB: Institutional Review Board; CVA: cerebrovascular accident; COPD: chronic obstructive pulmonary disease; SDs: standard deviations; HRs: hazard ratios

## Data Sharing Statement

The dataset used in this study is held by the Taiwan Ministry of Health and Welfare (MOHW). The Ministry of Health and Welfare must approve our application to access this data. Any researcher interested in accessing this dataset can submit an application form to the Ministry of Health and Welfare requesting access. Please contact the staff of MOHW (Email: stcarolwu@mohw.gov.tw) for further assistance. Taiwan Ministry of Health and Welfare Address: No.488, Sec. 6, Zhongxiao E. Rd., Nangang Dist., Taipei City 115, Taiwan (R.O.C.). Phone: +886-2-8590-6848. All relevant data are within the paper.
